# Healthcare-associated viral respiratory infections at a Canadian tertiary pediatric hospital: a seven-year retrospective analysis

**DOI:** 10.1017/ash.2024.452

**Published:** 2024-11-14

**Authors:** Sarah L Silverberg, Megan Clarke, Laurie Streitenberger, Kevin Brown, Aaron Campigotto, Michelle Science

**Affiliations:** 1 Department of Paediatrics, Division of Infectious Diseases, Hospital for Sick Children, Toronto, ON, Canada; 2 Infection Prevention and Control, Hospital for Sick Children, Toronto, ON, Canada; 3 Public Health Ontario, Toronto, ON, Canada; 4 Department of Paediatric Laboratory Medicine, Division of Microbiology, Hospital for Sick Children, Toronto, ON, Canada

## Abstract

Healthcare-associated viral respiratory infections (HA-VRIs) in a pediatric hospital decreased from 1.44 per 1000 patient days in 2019–0.43 and 0.38 in 2020–2021 during the SARS-CoV-2 pandemic but increased to 1.35 in 2022. The increase in HA-VRIs in 2022 coincided with the rise in community circulation of these organisms.

## Background

Rates of healthcare-associated viral respiratory infections (HA-VRIs) in pediatric patients have been shown to mirror community incidence with increased rates during viral respiratory season.^1,2^ After the onset of the SARS-CoV-2 pandemic, increased hospital infection control practices (ICP) and community public health measures were implemented to curb transmission rates; as a result, there were lower rates of respiratory pathogens circulating in the community.^3–5^ This study aims to review the incidence of HA-VRIs since 2016 and review the impact of hospital ICP on HA-VRI incidence during the pandemic period.

## Methods

We conducted a retrospective review of all HA-VRIs at the Hospital for Sick Children, in Toronto Canada, from January 1, 2016, through December 31, 2022. To define a HA-VRI, patients needed at least one respiratory symptom and laboratory confirmation of a virus from a respiratory specimen. Symptom onset had to occur on or after the third day of hospitalization, or within three days after discharge (see supplementary methods for additional details). Viral testing was done by molecular methods and became the primary modality of testing in 2016. Molecular testing did not differentiate enterovirus from rhinovirus until the beginning of 2020.

### Infection prevention and control (IPAC) measures

Enhanced IPAC precautions were implemented in March 2020 including universal masking hospital-wide and restricted numbers of in-hospital through the study period (see supplementary methods). Influenza vaccinations for staff were strongly recommended and SARS-CoV-2 vaccination was mandated in fall 2021. All patients with respiratory symptoms were placed on droplet and contact precautions regardless of etiology (with an N95 respirator for suspected or confirmed SARS-CoV-2); precautions were continued for prespecified periods depending on organism identified, otherwise remained on precautions while symptomatic. Before 2020, bedside nurses were responsible for screening visitors. Although policy before 2020, since the onset of the COVID-19 pandemic, all staff who were ill were specifically instructed to stay home. Screeners at hospital doors were present from March 2020 until May 9 2023 to monitor patients and caregivers for respiratory symptoms. From May 10 2023, only children under age twelve were actively screened.

### Statistical analysis

We calculated the overall HA-VRI rates per 1000 patient-days ([number of HA-VRI episodes/number of patient-days] × 1000). Patient days were calculated by unit and month.

Aggregated annual HA-VRI rates were compared using a Poisson regression generalized linear model including all years studied categorically. We conducted further analyses categorically defining pandemic years as 2020 and 2021 and adjusting for these in analyses that evaluated HA-VRI rates by year. Statistical significance was determined using 2-sided *P* values (*P* < .05). Analyses were completed using the R software environment (R [Version 4.1.3; The R Project for Statistical Computing, Vienna, Austria]).

## Results

**Figure 1.**
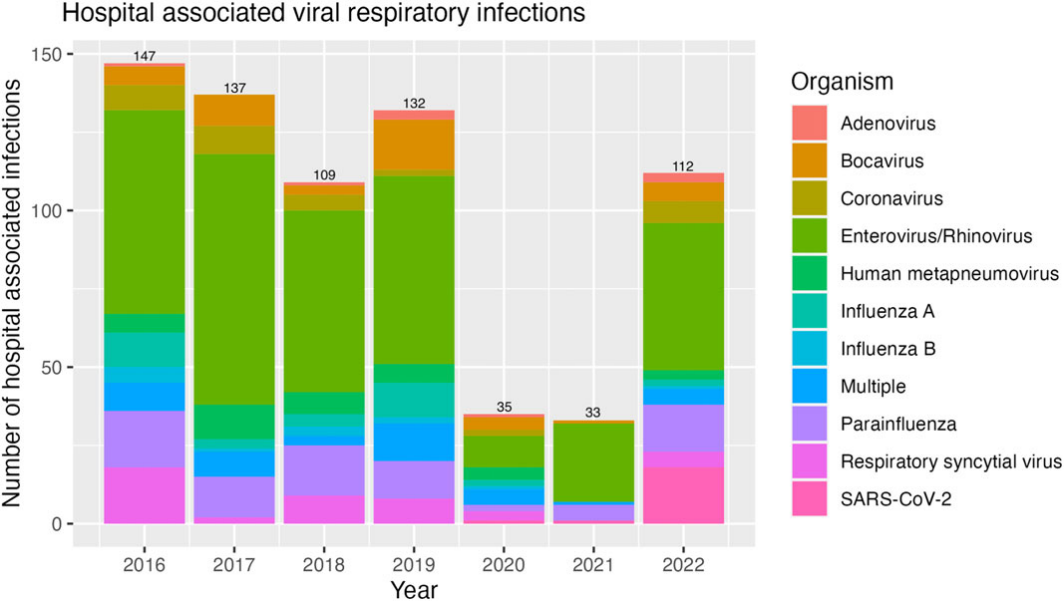
Hospital-associated viral respiratory infections per year of infection^1,2^. Total number of hospital associated viral respiratory infections by year and organism. Total infections per year denoted at top of each column; rhinovirus and enterovirus were not able to be distinguished by the lab assay used 2016–2020.

Over the study period 2016–2022, 705 HA-VRIs were documented over 623,000 patient days, of which there were 345 rhinovirus/enterovirus, 81 parainfluenza, 45 RSV, 33 influenza A, 13 influenza B, 20 SARS-CoV-2, 43 co-infections; 168 were a combination of adenovirus, bocavirus, seasonal coronavirus, and human metapneumovirus (Table 1, Figure 1). There was a significant reduction in HA-VRIs in 2020 and 2021, when enhanced precautions were implemented part-way through the 2020 peak viral season (35 and 33 HA-VRIs respectively compared to an average of 131 per year between 2016 and 2019).

**Table 1.** Patient characteristics

Characteristic (n = 705)	n (%)
**Year**	
2016	147 (14.0)
2017	137 (13.0)
2018	109 (10.4)
2019	132 (12.5)
2020	35 (3.3)
2021	33 (3.1)
2022	112 (10.6)
**Season**	
March-May	184 (26.1)
June-August	138 (19.6)
September-November	204 (28.9)
December-February	179 (25.4)
**Organism**	
Adenovirus	9 (1.3)
Bocavirus	46 (6.5)
Seasonal Coronavirus	33 (4.7)
SARS-CoV-2	20 (2.8)
Enterovirus/Rhinovirus	345 (48.9)
Human metapneumovirus	37 (5.3)
Influenza A and B	46 (6.5)
Parainfluenza	81 (11.5)
Respiratory Syncytial Virus	45 (6.4)
Multiple viruses	43 (6.1)
**Unit**	
Critical Care	78 (11.1)
NICU	19 (2.7)
General Pediatrics	99 (14.0)
Mixed medical and surgical	211 (29.9)
Surgical	150 (21.3)
Hematology/oncology and bone marrow transplant	168 (23.8)
**Age Distribution**	
0–11 months	331 (47.0)
1–5 years	232 (32.9)
6–11 years	78 (11.1)
12 and older	64 (9.1)
**Day of Admission at Infection**	
3 days	1 (0.1)
4–7 days	107 (15.2)
8–14 days	153 (21.7)
15–28 days	127 (18.0)
>28 days	317 (45.0)

HA-VRI rates demonstrated a reduction in HA-VRIs in the summer across all years and elevated rates of infection October-May (Supplementary Figure 1). Children were admitted for a mean of 55 days prior to infection (SD: 71.8); 317 (45.0%) took place after 28 days in hospital (Table 1). Almost half (331, 47.0%) of the HA-VRIs were found in those under age one, while another 232 (32.9%) took place in those ages 1–5; only 142 (20%) took place in those ages six and above (Table 1).

There was an increased incidence of 1.65 per 1000 patient days on the mixed medical/surgical unit compared to general pediatrics (RR: 3.72, 95% CI: 2.94, 4.74) and on hematology/oncology compared to general pediatrics with 1.38 HA-VRI per 1000 patient days (RR: 2.49, 95%CI: 1.94, 3.20). There was a low incidence in the neonatal ICU (NICU) (0.2 per 1000 patient days) compared to all other units (RR: 0.29, 95%CI: 0.17, 0.47).

Children under age one had higher rates of infections after more than 28 days in hospital than other ages (*P* < 0.001). Despite the high proportion of infants who developed a HA-VRI, few were in the NICU (5.7%) even though the NICU represented 14% of patient days, and the high proportion of infants who developed a HA-VRI after >4 weeks in hospital were overwhelmingly non-NICU patients (97.5%).

### SARS-CoV-2 pandemic measures (implemented March 2020)

There was an overall incidence of HA-VRIs of 1.13 per 1000 patient days across the study period. Although the yearly incidence of HA-VRIs between 2016 and 2019 ranged from 1.19 to 1.56 per 1000 patient days, there was a significant reduction to 0.43 HA-VRIs per 1000 patient days in 2020 and 0.38 in 2021, with a RR in 2020 of 0.3 compared to 2016–2019 (95% CI: 0.21, 0.42), and RR in 2021 of 0.27 compared to 2016–2019 (95% CI: 0.19, 0.38). Although there was no change in enhanced IPAC precautions in 2022 compared to 2021, HA-VRIs increased in 2022 with an annual incidence of 1.35 in 2022 (RR 0.95 compared to 2016–2019, 95% CI: 0.77, 1.16, and 3.52 compared to 2021, 95% CI: 2.42, 5.27).

## Discussion

Although there was a significant decrease in HA-VRIs during 2020 and 2021 reflecting the onset of the COVID-19 pandemic, there were increased HA-VRIs in 2022 despite minimal changes to enhanced IPAC procedures between 2021 and 2022. Although others have reported similar findings of decreased HA-VRIs during the COVID-19 pandemic, we believe this is the first to demonstrate the rise later in the pandemic despite ongoing enhanced ICP in the hospital.^7,8^ Our data therefore suggest that the decreased rates in 2020–2021 may be reflective of the overall decrease in community burden of respiratory illness. For example, we saw a decreased incidence in RSV, influenza and human metapneumovirus in these years which reflects similar community circulation incidence in 2020–2021; similar to the community, we also observed a rise in 2022 HA-VRIs.^9^ Alternatively, the rise of HA-VRIs we observed in 2022 could represent a decrease in adherence of healthcare workers and families to enhanced infection precautions over time. Further investigations into the perception of and adherence to ICP, particularly in a pandemic setting, could better explore these possibilities.

Although our data does support the historical pattern of increased viral respiratory infections over the winter, we continue to see sustained nosocomial HA-VRI even at times of lower community circulation. This persistent circulation challenges the seasonal implementation of enhanced hospital measures to reduce spread of HA-VRIs and supports the consideration of year-round measures.

Our study has limitations. As our study only reflects nosocomial infections that would require symptoms to prompt testing, we may not have captured all pauci-symptomatic or asymptomatic infections. Our data also do not reflect cases in healthcare workers and/or family members who may have acquired a HA-VRI. Further, we did not collect information on the adherence to the enhanced hospital ICP outside of standard patient safety measures (ie, hand hygiene), to better understand whether this played a role in the increase in HA-VRIs in 2022. Finally, we did not separate out these two months from the remainder of the year; thus, the relative rates of HA-VRI in 2020 may be slightly higher than those after the introduction of such measures.

## Supporting information

Silverberg et al. supplementary material 1 Silverberg et al. supplementary material

Silverberg et al. supplementary material 2 Silverberg et al. supplementary material

Silverberg et al. supplementary material 3 Silverberg et al. supplementary material

Silverberg et al. supplementary material 4 Silverberg et al. supplementary material

## Supplementary material

The supplementary material for this article can be found at https://doi.org/10.1017/ash.2024.452.

## References

[1] Quach C , Shah R , Rubin LG. Burden of healthcare-associated viral respiratory infections in children’s hospitals. J Pediatr Infect Dis Soc 2016;7:18–24. 10.1093/jpids/piw072 PMC7204516 28040689

[2] Northway T , Langley JM , Skippen P. Health care–associated infection in the pediatric intensive care unit: epidemiology and control—keeping patients safe. Pediatr Critic Care 2011:1349.

[3] Groves HE , Piché-Renaud P-P , Peci A , et al. The impact of the COVID-19 pandemic on influenza, respiratory syncytial virus, and other seasonal respiratory virus circulation in Canada: A population-based study. Lancet Region Health Am 2021;1:100015. 10.1016/j.lana.2021.100015 PMC8285668 34386788

[4] Poole S , Brendish NJ , Clark TW. SARS-CoV-2 has displaced other seasonal respiratory viruses: Results from a prospective cohort study. J Infect 2020;81:966–972. 33207254 10.1016/j.jinf.2020.11.010 PMC7666810

[5] Peci A , Tran V , Guthrie JL , et al. Prevalence of co-infections with respiratory viruses in individuals investigated for SARS-CoV-2 in Ontario, Canada. Viruses 2021;13:130. 33477649 10.3390/v13010130 PMC7831481

[6] Health Quality Ontario. *Hand Washing Hosp Care Provid* 2024.

[7] Lefebvre M-A , Rajda E , Frenette C , et al. Impact of the COVID-19 pandemic on healthcare-associated viral respiratory infections at a tertiary care pediatric hospital. Am J Infect Control 2023;51:961–963. 36736901 10.1016/j.ajic.2023.01.017 PMC9889274

[8] Wee L , Conceicao E , Sim X , Ko K , Ling M , Venkatachalam I. Reduction in healthcare-associated respiratory viral infections during a COVID-19 outbreak. Clin Microbiol Infect 2020;26:1579–1581. 32622953 10.1016/j.cmi.2020.06.027 PMC7332451

[9] Public Health Agency of Canada. Respiratory Virus Report, Week 38 - ending September 24, 2022. In: Health Mo, ed2022. Accessed September 20, 2024.

