# Supplementary figures and images for: Healthcare-associated viral respiratory infections at a Canadian tertiary pediatric hospital: a seven-year retrospective analysis
Source: Antimicrob Steward Healthc Epidemiol. 2024 Nov 14;4(1):e205. doi: 10.1017/ash.2024.452 (PMC11574598; doi:10.1017/ash.2024.452)

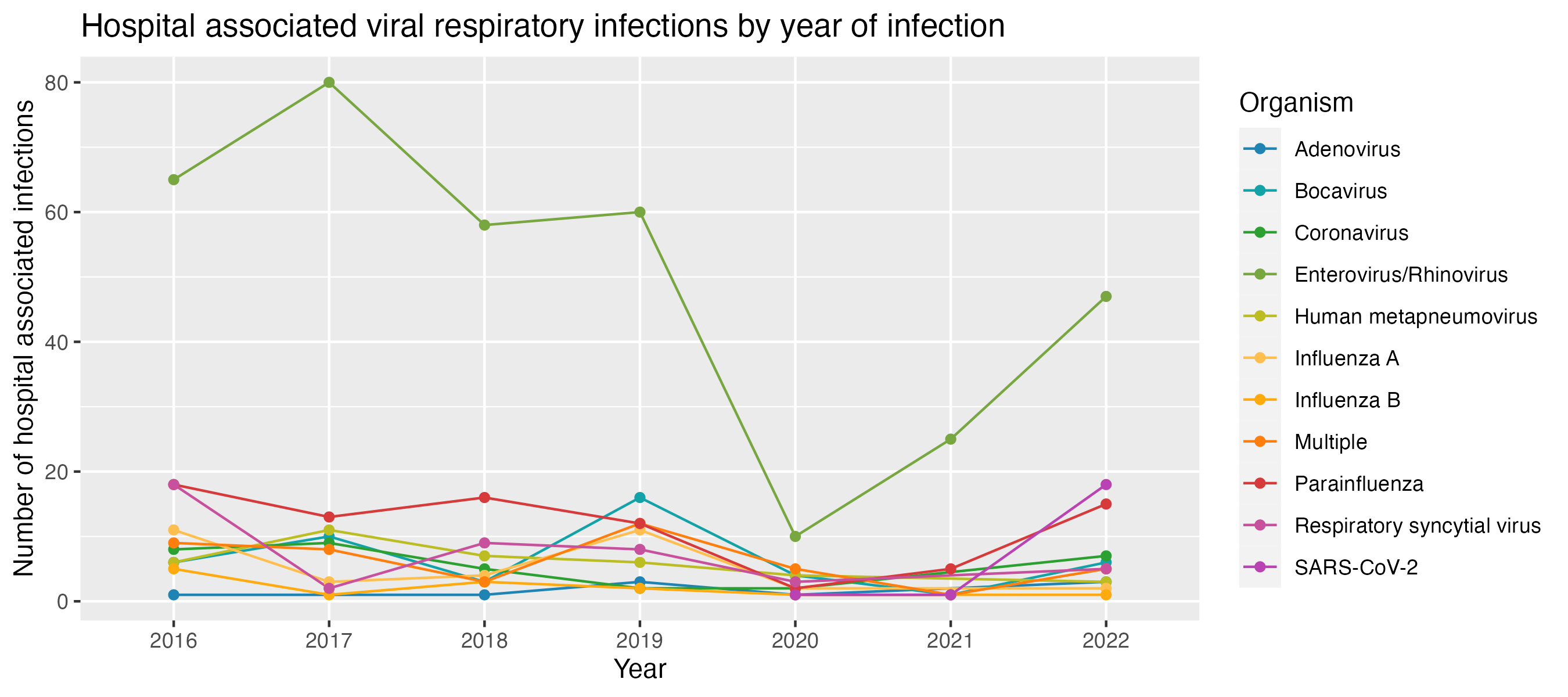

Supplement: Silverberg et al. supplementary material 1 — Silverberg et al. supplementary material [file S2732494X24004522sup001.tiff]

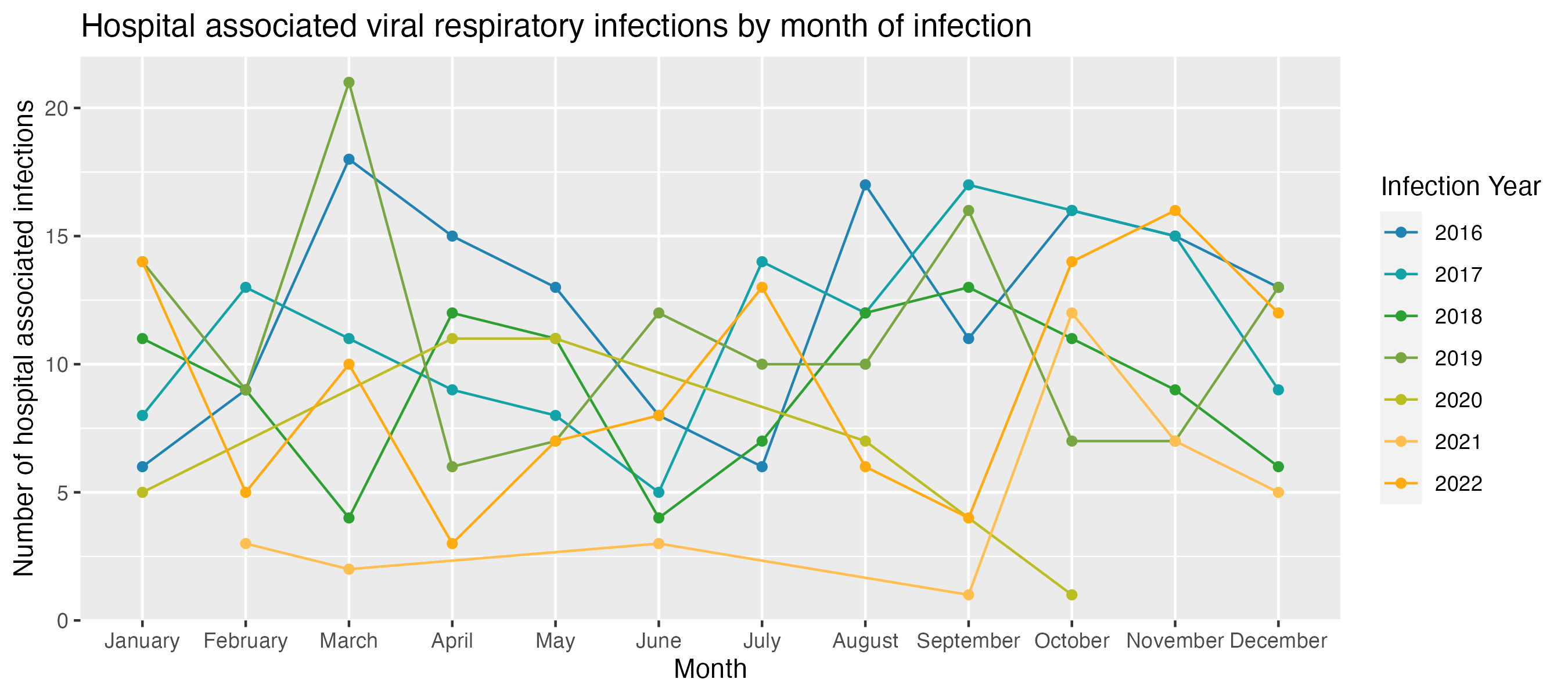

Supplement: Silverberg et al. supplementary material 2 — Silverberg et al. supplementary material [file S2732494X24004522sup002.tiff]

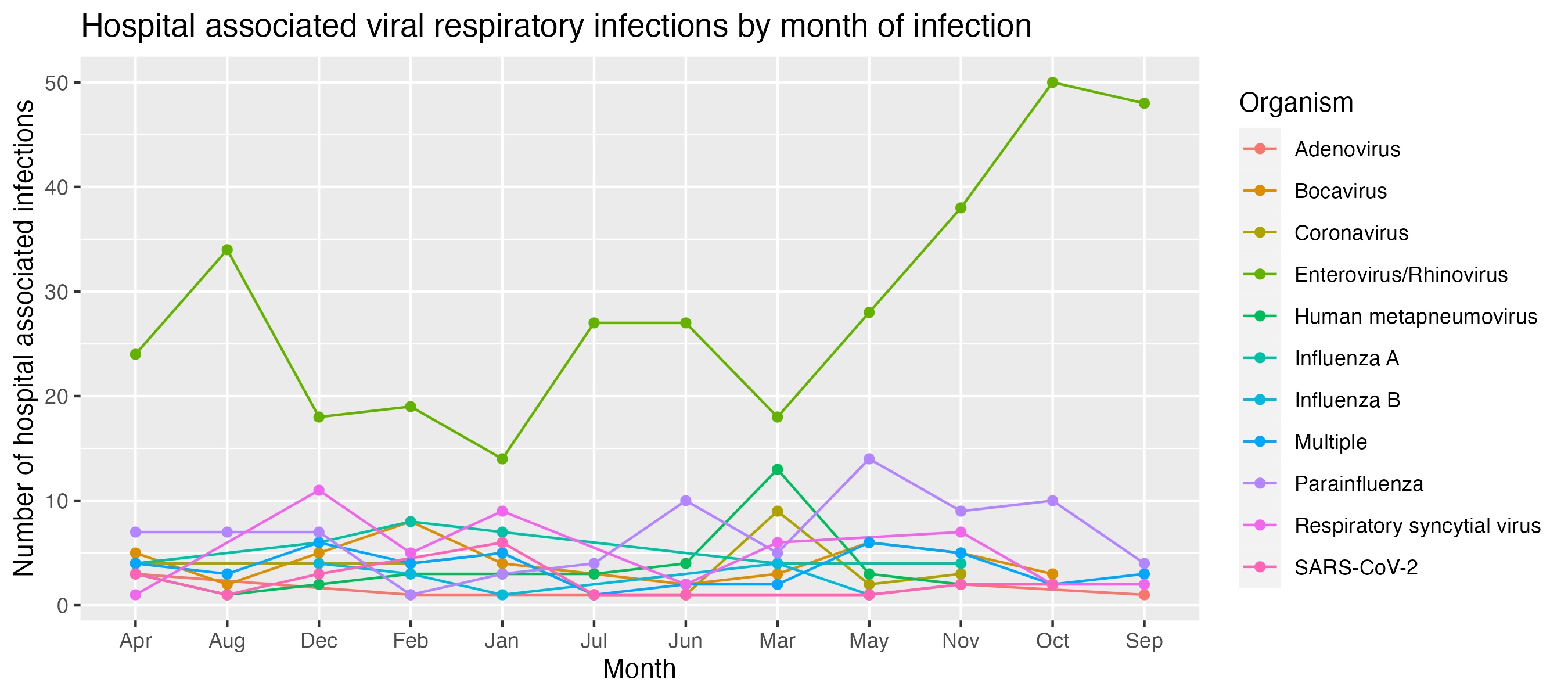

Supplement: Silverberg et al. supplementary material 3 — Silverberg et al. supplementary material [file S2732494X24004522sup003.zip › Supplementary Figure in Manuscript (1).jpg]
