# Supplementary material for: Healthcare-associated viral respiratory infections at a Canadian tertiary pediatric hospital: a seven-year retrospective analysis
Source: Antimicrob Steward Healthc Epidemiol. 2024 Nov 14;4(1):e205. doi: 10.1017/ash.2024.452 (PMC11574598; doi:10.1017/ash.2024.452)
Supplement: Silverberg et al. supplementary material 4 — Silverberg et al. supplementary material [file S2732494X24004522sup004.docx]

**Supplementary Methods**

*Additional case definition criteria*

A patient positive for more than one virus was considered a single HA-VRI. If they tested positive for the same virus multiple times during admission, it was only considered a new infection if they had onset of new respiratory symptoms and a clear resolution of their initial infection.

*Additional statistical analyses*

Patient days were not calculated for one unit (5D) until 2019 as it was previously used only as a short-stay unit, for which patient days were not tracked. In addition to aggregate annual HA-VRI rates, HA-VRI rates by unit were compared using a Poisson regression generalized linear model including all units.

*Additional infection prevention and control measures*

Most patients outside of intensive care units (ICUs) were in single patient rooms; within ICUs, patients with viral respiratory symptoms were preferentially placed in single rooms, and occasionally cohorted by organism when necessary. Hand hygiene compliance rose from 85% at the beginning of the study period to 93-98% throughout the rest of the study period.^6^ Visitor restrictions included restriction of siblings being allowed in hospital (with exceptions made case-by-case).
